# Identifying Fishes through DNA Barcodes and Microarrays

**DOI:** 10.1371/journal.pone.0012620

**Published:** 2010-09-07

**Authors:** Marc Kochzius, Christian Seidel, Aglaia Antoniou, Sandeep Kumar Botla, Daniel Campo, Alessia Cariani, Eva Garcia Vazquez, Janet Hauschild, Caroline Hervet, Sigridur Hjörleifsdottir, Gudmundur Hreggvidsson, Kristina Kappel, Monica Landi, Antonios Magoulas, Viggo Marteinsson, Manfred Nölte, Serge Planes, Fausto Tinti, Cemal Turan, Moleyur N. Venugopal, Hannes Weber, Dietmar Blohm

**Affiliations:** 1 Centre for Applied Gene Sensor Technology (CAG), FB2-UFT, University of Bremen, Bremen, Germany; 2 Marine Biology, Free University of Brussels (VUB), Brussels, Belgium; 3 Institute of Marine Biology and Genetics, Hellenic Centre of Marine Research, Heraklion, Crete, Greece; 4 Department of Functional Biology, Universidad de Oviedo, Oviedo, Spain; 5 Department of Experimental Evolutionary Biology, University of Bologna, Bologna, Italy; 6 UMR 5244 CNRS-EPHE-UPVD, Université de Perpignan, Perpignan, France; 7 Prokaria, Matis ohf, Reykjavik, Iceland; 8 Faculty of Life and Environmental Sciences, University of Iceland, Reykjavík, Iceland; 9 Zentrum für Technomathematik (ZeTeM), University of Bremen, Bremen, Germany; 10 Faculty of Fisheries, Mustafa Kemal University, Iskenderun, Hatay, Turkey; 11 College of Fisheries, Mangalore, Karnataka, India; American Museum of Natural History, United States of America

## Abstract

**Background:**

International fish trade reached an import value of 62.8 billion Euro in 2006, of which 44.6% are covered by the European Union. Species identification is a key problem throughout the life cycle of fishes: from eggs and larvae to adults in fisheries research and control, as well as processed fish products in consumer protection.

**Methodology/Principal Findings:**

This study aims to evaluate the applicability of the three mitochondrial genes 16S rRNA (16S), cytochrome *b* (cyt *b*), and cytochrome oxidase subunit I (COI) for the identification of 50 European marine fish species by combining techniques of “DNA barcoding” and microarrays. In a DNA barcoding approach, neighbour Joining (NJ) phylogenetic trees of 369 16S, 212 cyt *b*, and 447 COI sequences indicated that cyt *b* and COI are suitable for unambiguous identification, whereas 16S failed to discriminate closely related flatfish and gurnard species. In course of probe design for DNA microarray development, each of the markers yielded a high number of potentially species-specific probes *in silico*, although many of them were rejected based on microarray hybridisation experiments. None of the markers provided probes to discriminate the sibling flatfish and gurnard species. However, since 16S-probes were less negatively influenced by the “position of label” effect and showed the lowest rejection rate and the highest mean signal intensity, 16S is more suitable for DNA microarray probe design than cty *b* and COI. The large portion of rejected COI-probes after hybridisation experiments (>90%) renders the DNA barcoding marker as rather unsuitable for this high-throughput technology.

**Conclusions/Significance:**

Based on these data, a DNA microarray containing 64 functional oligonucleotide probes for the identification of 30 out of the 50 fish species investigated was developed. It represents the next step towards an automated and easy-to-handle method to identify fish, ichthyoplankton, and fish products.

## Introduction

World fishery production (capture fisheries and aquaculture) reached 143.6 million tons in 2006, 77% of which were used for human consumption. About 37% of the total production entered the international trade, with an import value of up to 62.8 billion Euro in 2006. Europe produces about 15.5 million tons of fish and fishery products per year, an amount that is insufficient to satisfy the demand. The import value of fish and fishery products for Europe reached about 28 billion Euro in 2006, comprising 44.6% of the global imports. Trading within the European Union (EU) is extremely important, because about 45% of imports and 84% of exports are being conducted between EU countries [1]. These figures underline the importance of the global trade in fish and fishery products, especially for the EU.

In order to protect the consumer, the EU has strict regulations for seafood labelling, which must include the species name (EU Council Regulation No 104/2000, EU Commission Regulation No 2065/2001). However, detection of commercial fraud by mislabelling is difficult, especially in processed products, where all morphological characters suitable for species identification have been eliminated. Furthermore, the large number of traded species from all over the world, e.g. 420 fish species in Germany, is making it impossible for the inspection authorities to control for correct labelling. The genetic identification of species can help to solve this problem [2]–[4]. For instance, a DNA sequencing study on food fish has revealed that three-quarters of fish sold in the United States of America as “red snapper” were mislabelled and belonged to other species [5]. Mislabelling can even threaten consumer health if toxic species enter the market, such as pufferfish that causes tetrodotoxin poisoning [6].

Accurate species identification is also essential in ichthyoplankton surveys for fisheries research, conducted to estimate stock of future year-classes and to fix fishing quota accordingly. For instance, eggs of cod, haddock, and whiting are difficult to differentiate by morphological characters. Genetic identification revealed that almost two thirds of “cod like” eggs from the Irish Sea have been misidentified, resulting in an overestimation of cod stocks [7].

Mitochondrial DNA (mtDNA) sequences of cytochrome *b* (cyt *b*) and 16S rRNA (16S) genes are amongst the most widely used genetic markers for fish species identification [3], [4]. They have been widely applied in seafood control [5], [8]–[11], ichthyoplankton identification [12]–[15], fisheries control [16], [17], and species delineation [18]–[20]. Data bases have been established, containing complete cyt *b* and rhodopsin gene sequences of European marine fish species [21] (www.fishtrace.org), as well as partial 16S, cyt *b*, and COI sequences of anchovies [11] (http://anchovyid.jrc.ec.europa.eu) to enable a sequence-based identification of specimens.

However, in course of developing a unifying identification system for animal species an universal marker has been proposed to serve as a so-called “DNA barcode” [22], [23]. This DNA barcode is the sequence of the “Folmer fragment” [24], a polymorphic part of the mitochondrial cytochrome oxidase subunit I gene (COI), which can be used to identify closely related species as well as higher taxa in many animal phyla. The applicability of COI for species identification in fish [25] triggered actually the international initiative for barcoding all fishes (FISH-BOL; www.fishbol.org) [3], [26]. Additional studies have shown that genetic identification by “COI barcodes” can provide a useful tool to identify seafood for consumer protection [9], [27]–[30], to control fisheries [31]–[33], to detect possibly cryptic species [34]–[37], and even to describe new species [38].

DNA sequence-based identification utilises the refined Sanger sequencing method [39], [40], which is still the “gold standard” [41], but requires samples that contain DNA of only one specimen. However, this is not the case in ichthyoplankton or other mixed samples, where several target species need to be detected and discriminated amongst an even much higher number of other species. Most next generation sequencing methods are enabling the analysis of mixed samples, but need highly sophisticated and expensive equipment (for review see e.g. [3] and references therein).

In contrast, DNA microarrays, first created 20 years ago, are well established and able to differentiate hundreds of specimens simultaneously. They were primarily used for gene expression profiling, but recently several DNA microarrays have been developed for the identification of fishes [42]–[45] and other organisms (see references in [3]).

This study compares three genetic markers (16S, cyt *b*, and COI) used as identification tools to distinguish 50 fish species common in European seas in terms of (1) their power of resolution in sequence-based species identification (DNA barcoding) and (2) their applicability in oligonucleotide probe design for the development of a low density DNA microarray.

## Materials and Methods

### Sampling and DNA Extraction

**Figure 1:**
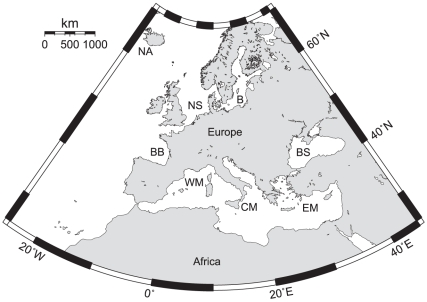
Map with sampling areas for fishes from European seas. Northeastern Atlantic (NA), North Sea (NS), Baltic (B), Bay of Biscay (BB), Western Mediterranean (WM), Central Mediterranean (CM), Eastern Mediterranean (EM), and Black Sea (BS).

In order to account for intraspecific sequence variation and to avoid any misleading results due to restricted sampling in terms of specimens and geographic coverage [46], fishes were collected in eight different regions of the European seas: Northeastern Atlantic, North Sea, Baltic, Bay of Biscay, Western, Central, as well as Eastern Mediterranean, and Black Sea (Fig. 1, Table 1, Supporting Information Table S1). Taxonomic sampling focused on commercially important species such as anchovy, cod, flounder, hake, herring, plaice, sardine, and sole. However, considering that differentiation of closely related species constitutes a challenging task not only for morphological but genetic methods as well, the sampling scheme also included a number of sibling species and groups of closely related fishes that are commercially not important, in order to examine the resolution power of the markers in species delineation.

**Table 1:** Sequences utilised for the DNA barcoding approach.

Species	Family	Order	16S	cyt *b*	COI	Total
*Clupea harengus*	Clupeidae	C	**2**	**3**	**5**	**10**
*Sardina pilchardus*	Clupeidae	C	**11**	**7**		**18**
*Engraulis encrasicolus*	Engraulidae	C	**11**	**8**	**12**	**31**
*Gadus morhua*	Gadidae	G	**5**	**5**	**5**	**15**
*Merlangius merlangus*	Gadidae	G	**4**	**4**	**4**	**12**
*Merluccius merluccius*	Merlucciidae	G	**14**	**2**	**19**	**35**
*Lophius budegassa*	Lophiidae	L	**8**	**1**	**6**	**15**
*Lophius piscatorius*	Lophiidae	L	**4**	**2**	**3**	**9**
*Trachurus mediterraneus*	Carangidae	P	**3**	**6**	**12**	**21**
*Trachurus picturatus*	Carangidae	P	**5**	**5**	**6**	**16**
*Trachurus trachurus*	Carangidae	P	**9**	**13**	**12**	**34**
*Dicentrarchus labrax*	Moronidae	P	**5**	**1**	**8**	**14**
*Mullus barbatus*	Mullidae	P	**10**	**12**	**12**	**34**
*Mullus surmuletus*	Mullidae	P	**14**	**13**	**3**	**30**
*Scomber japonicus*	Scombridae	P	**9**	**3**	**17**	**29**
*Scomber scombrus*	Scombridae	P	**4**	**2**	**11**	**17**
*Epinephelus marginatus*	Serranidae	P	**8**	**0**	**5**	**13**
*Serranus cabrilla*	Serranidae	P	**8**	**6**	**15**	**29**
*Serranus hepatus*	Serranidae	P	**8**	**6**	**5**	**19**
*Serranus scriba*	Serranidae	P	**6**	**1**	**5**	**12**
*Boops boops*	Sparidae	P	**9**	**7**	**23**	**39**
*Diplodus sargus*	Sparidae	P	**5**	**4**	**4**	**13**
*Diplodus vulgaris*	Sparidae	P	**8**	**7**	**22**	**37**
*Pagellus acarne*	Sparidae	P	**8**	**9**	**12**	**29**
*Pagellus erythrinus*	Sparidae	P	**10**	**7**	**15**	**32**
*Sparus aurata*	Sparidae	P	**7**	**6**	**11**	**24**
*Arnoglossus laterna*	Bothidae	Pl	**5**	**0**	**11**	**13**
*Hippoglossoides platessoides*	Pleuronectidae	Pl	**2**	**0**	**3**	**5**
*Limanda limanda*	Pleuronectidae	Pl	**11**	**3**	**6**	**20**
*Microstomus kitt*	Pleuronectidae	Pl	**2**	**3**	**4**	**9**
*Platichthys flesus*	Pleuronectidae	Pl	**11**	**2**	**4**	**17**
*Pleuronectes platessa*	Pleuronectidae	Pl	**9**	**0**	**2**	**11**
*Lepidorhombus boscii*	Scophthalmidae	Pl	**12**	**5**	**9**	**26**
*Lepidorhombus whiffiagonis*	Scophthalmidae	Pl	**5**	**3**	**8**	**16**
*Phrynorhombus norvegicus*	Scophthalmidae	Pl	**3**	**3**	**3**	**9**
*Psetta maxima*	Scophthalmidae	Pl	**9**	**4**	**15**	**28**
*Scophthalmus rhombus*	Scophthalmidae	Pl	**9**	**8**	**13**	**30**
*Buglossidium luteum*	Soleidae	Pl	**5**	**0**	**13**	**18**
*Microchirus variegatus*	Soleidae	Pl	**4**	**2**	**9**	**15**
*Pegusa impar*	Soleidae	Pl	**3**	**0**	**0**	**3**
*Solea solea*	Soleidae	Pl	**15**	**0**	**18**	**33**
*Scorpaena notata*	Scorpaenidae	S	**11**	**5**	**10**	**26**
*Scorpaena porcus*	Scorpaenidae	S	**8**	**4**	**0**	**12**
*Helicolenus dactylopterus dactylopterus*	Sebastidae	S	**9**	**10**	**20**	**39**
*Chelidonichthys lucernus*	Triglidae	S	**10**	**11**	**16**	**37**
*Eutrigla gurnardus*	Triglidae	S	**3**	**1**	**2**	**6**
*Trigla lyra*	Triglidae	S	**6**	**0**	**1**	**7**
*Trigloporus lastoviza*	Triglidae	S	**7**	**0**	**5**	**12**
*Macrorhamphosus scolopax*	Centriscidae	Sy	**7**	**8**	**6**	**21**
*Zeus faber*	Zeidae	Z	**8**	**0**	**15**	**23**
	**Total**		**369**	**212**	**447**	**1023**

Abbreviations: 16S: 16S rRNA gene (accession numbers: FN687913–FN688280), cyt *b*: cytochrome *b* gene (accession numbers: FN688281–FN688492), COI: cytochrome oxidase subunit I gene (accession numbers: FN688905–FN689348), C: Clupeiformes, G: Gadiformes, L: Lophiiformes, P: Perciformes, Pl: Pleuronectiformes, S: Scorpaeniformes, Sy: Syngnathiformes, Z: Zeiformes. For details see Supporting Information Table S1.

Voucher specimens and tissue samples were preserved in absolute ethanol and were frozen at −20°C or stored at 4°C. DNA was extracted from muscle tissue with the DNeasy tissue kit (Qiagen, Hilden, Germany) or gill filaments with the Agowa mag midi DNA isolation kit (AGOWA, Berlin, Germany) according to the instructions of the manufacturers.

### Polymerase Chain Reaction and Sequencing

Three mitochondrial genes were screened as potential markers for species identification in this study: (1) 16S, (2) cyt *b*, and (3) COI. A fragment of 16S was amplified and sequenced as described in Kochzius et al. (2008) [42].

The cyt *b* fragment was amplified with the newly designed primers CytbF (5′-GGC TGA TTC GGA ATA TGC AYG CNA AYG G-3′) and CytbR (5′-GGG AAT GGA TCG TAG AAT TGC RTA NGC RAA-3′). Polymerase chain reaction (PCR) with a total volume of 15 µl contained 1.5 µl 10× reaction buffer, 1.5 µl dNTPs (10 mM), 0.05 µl of each primer (100 pmol/µl), 5 µl DNA-extract, 0.3 µl Teg polymerase (3 U/µl; comparable with Taq polymerase; Prokaria, Reykjavik, Iceland), and 6.6 µl deionised ultra-pure water. Thermal profile began at 94°C for 4 min, followed by 35 cycles of 94°C (30 s), 52°C (30 s), and 72°C (90 s), with a final step of 7 min at 72°C.

In order to amplify a fragment of COI, degenerated primers were designed on the basis of the universal COI primers for fish published by Ward et al. (2005) [25]: COI-Fish-F (5′-TTC TCA ACT AAC CAY AAA GAY ATY GG-3′) and COI-Fish-R (5′-TAG ACT TCT GGG TGG CCR AAR AAY CA-3′. The volume of the PCRs was 15 µl and contained 1.5 µl 10× reaction buffer, 1.5 µl dNTPs (10 mM), 0.05 µl of each primer (100 pmol/µl), 3 µl DNA-extract, 0.2 µl Teg polymerase (3 U/µl; Prokaria, Reykjavik, Iceland), and 9.7 µl deionised water. Thermal profile started with 94°C for 4 min, followed by 30 cycles of 94°C (50 s), 59°C (50 s), and 72°C (90 s), finalised at 72°C for 7 min.

PCR products were purified by using the ExoSAP-IT for PCR clean-up (GE Healthcare, Uppsala, Sweden). The newly designed sequencing primer cytbFseq (5′- GGC TGA TTC GGA ATA TGC A-3′) was used to sequence the cyt *b* PCR products. The COI product were sequenced with the PCR primers shown above. The BigDye Terminator Cycle Sequencing Kit (ver. 3.1, PE Biosystems, Foster City, USA) and an ABI Prism 3730 automated DNA Analyser (Applied Biosystems, Foster City, USA) were used according to the manufacturer's instructions.

### DNA Barcoding

Sequences of 50 marine fish species were obtained to compare the applicability of the 16S, cyt *b*, and COI genes as markers for DNA barcoding. Multiple alignments of these orthologous sequences were performed with the programme Clustal W [47] as implemented in BioEdit (version 7.0.4.1) [48] to ensure that all sequences of each marker gene provide a homologous fragment. Cytochrome *b* and COI sequences were translated into amino acids with the program Squint (www.cebl.auckland.ac.nz) in order to exclude sequencing errors and to avoid the inclusion of pseudogene sequences in the datasets. For each marker, unrooted Neighbour Joining (NJ) trees were constructed and genetic *p*-distance was calculated within species, genera, families, and orders with the programme MEGA (version 3.1) [49]. Evaluation of statistical confidence in nodes was based on 1000 non-parametric bootstrap replicates [50]. Since the aim of this task was to identify species using barcodes, phylogenetic trees were constructed without selecting *a priori* an evolutionary model appropriate for the dataset.

### *In Silico* Oligonucleotide Probe Design

The design of oligonucleotide probes was based on sequence alignments used for DNA barcoding that also included additional sequences obtained from international sequence data bases: 35 for 16S, 69 for cyt *b*, and 23 for COI. Gaps in the 16S sequence alignment were removed before probe design. Species-specific oligonucleotide probes that cover all sequences of one species and do not match any other species were designed with a computer programme developed by the bioinformatics groups of the Centre for Applied Gene Sensor Technology (CAG) and the Zentrum für Technomathematik (ZeTeM), both at University of Bremen [51]. Probe design was performed in order to meet the following criteria: (1) optimal length of 23 to 27 bp, (2) melting temperature (T_m_) of 81 to 85°C based on the unified model [52], (3) GC content of 52% to 54%, (4) appropriate secondary structure of the oligonucleotides and the target sequence, (5) possible dimer formation, and (6) a suitable probe-target binding energy. The programme RNAfold [53] was employed to compute minimal free energy structures. Probes showing strong secondary structures or binding to a region of the target with such a strong secondary structure were not used. The selected oligonucleotide probes were tested *in silico* against >900 16S (365 species), >2700 cyt *b* (324 species), and >270 COI (93 species) sequences of fishes occurring in European seas. These sequences were obtained from EMBL sequence data base (92%) and were sequenced in course of this study (8%).

### Preparation of DNA Microarrays and Hybridisation Experiments

Glass slides coated with aminosilane (3-aminopropyltrimethoxysilane) and a PDITC-linker (1,4-phenylendiisothiocyanate) (Asper Biotech, Tartu, Estonia) were used for microarray production. A spotting robot based on a modified version of the contactless TopSpot® technology [54] was used to spot oligonucleotide probes (Thermo Hybaid, Ulm, Germany) with a 5′-amino-C6-modification in 150 mM Na_3_PO_4_ buffer (pH 8.5) at a concentration of 20 µM onto the glass slides. The spotted volume of this oligonucleotide solution was 200 pl, producing a spot diameter of approximately 220 µm. Afterwards, the microarrays were incubated for 16 h in a wet chamber to ensure efficient covalent binding of the oligonucleotides to the surface. Finally, the microarrays were shrink-wrapped under a nitrogen atmosphere and were stored at 4°C for up to 6 months. Each probe was spotted in three replicates.

DNA of the 50 target fish species (Table 1) was separately amplified and labelled with 5′-Cy5-modified primers for single target hybridisation experiments. A fragment of 16S was amplified and labelled as described in Kochzius et al. (2008) [42].

Labelled cyt *b* fragments of 626 bp length were PCR amplified with the 5′-Cy5-modified primers CytbF and CytbR. Reactions were conducted in a volume of 100 µl containing 10 µl 10× reaction buffer, 8 µl MgCl_2_ (50 mM), 4 µl dNTPs (5 mM), 4 µl of each primer (10 µM), 4 µl DNA-extract, 0.4 µl Taq polymerase (5 U/µl), 2 µl BSA (20 mg/ml), and 63.6 µl deionised water. The thermo-profile started at 94°C (2 min), followed by 40 cycles at 94°C (60 s), 45°C (90 s), and 72°C (60 s), finalised for 5 min at 72°C.

Amplification of labelled COI fragments of 710 bp length was performed with the 5′-Cy5-modified primer pair COI-Fish-F and COI-Fish-R. The PCR solution contained 10 µl 10× reaction buffer, 8 µl MgCl_2_ (50 mM), 4 µl dNTPs (5 mM), 4 µl of each primer (10 µM), 5 µl DNA-extract, 0.4 µl Taq polymerase (5 U/µl), 4 µl BSA (20 mg/ml), and 60.6 µl deionised water in a volume of 100 µl. Thermo-cycling did start at 94°C, with 35 subsequent cycles at 94°C (50 s), 45°C (50 s), and 72°C (90 s). The final step was 3 min at 72°C.

The Cy5-labelled PCR products were purified using the QIAquick PCR Purification Kit (QIAGEN, Hilden, Germany). Hybridisation experiments were performed with 50 target fish species (Table 1). The purified Cy5-labelled PCR product and a 5′-Cy3-labelled positive control (5′-CGT GTG AGT CGA TGG ATC ATA-3′) at concentrations of 10 and 1 nM, respectively, were hybridised to the microarray in a volume of 130 µl using GeneFrames® (ABgene House, Epsom, UK). Hybridisation was conducted at 50°C for 2 h in a hybridisation oven. Afterwards, GeneFrames® were removed and the microarrays were washed 5 minutes each with 2×SSC (sodium chloride trisodium citrate) buffer containing 0.05% SDS (sodium dodecyl sulphate), 1×SSC containing 0.05% SDS, and 1×SSC. Finally, the microarrays were dried in a centrifuge at 2000 rpm for 3 minutes. Each hybridisation experiment was conducted in three replicates.

### Measurement of Fluorescence Signals and Data Analysis

Hybridisation signals were measured using an Axon 4000B fluorescence microarray scanner at 635 nm (Cy5) and 528 nm (Cy3). The fluorescence signal analysis was conducted with the software GenePix 4.1 (Axon, Union City, USA). Spots that showed artefacts caused during the spotting process (e.g., inhomogeneous spots documented by a monitoring camera during spotting) or the experiment (e.g. air bubbles) were removed from the analysis. The fluorescence signal of each probe was measured as arbitrary units and the arithmetic mean was calculated. Only signals with a minimum value of 1000 arbitrary units were considered in data analysis.

## Results

### DNA Barcoding

A data set of 369 16S (418–452 bp; accession numbers FN687913–FN688280 ), 212 cyt *b* (404 bp; accession numbers FN688281–FN688492), and 447 COI (455 bp; accession numbers FN688905–FN689348) sequences of 50 fish species from European seas was obtained and these sequences are available at the EMBL sequence data base (Table 1, Supporting Information Table S1). No stop codons, insertions, and deletions were observed in the cyt *b* and COI sequences, indicating that they represent fragments of functional mitochondrial genes and not nuclear mitochondrial pseudogenes (Numts) [55].

**Figure 2:**
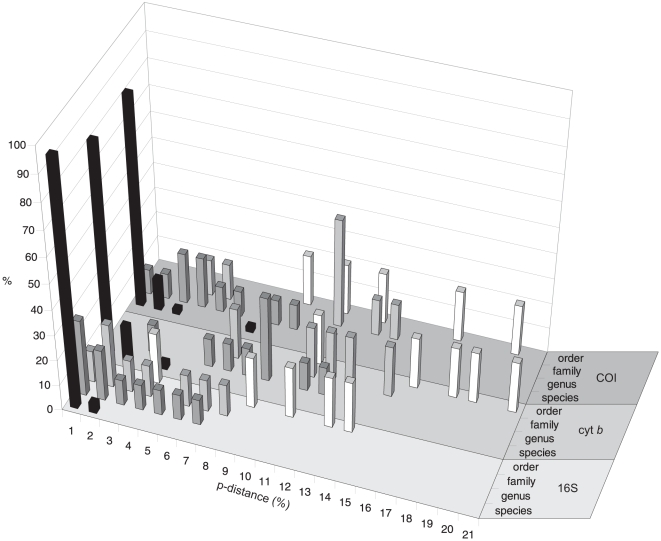
Frequency distribution of genetic p-distances. Data for different taxonomic levels for partial sequences from mitochondrial 16S rRNA (16S), cytochrome *b* (cyt *b*), and cytochrome oxidase subunit I (COI) genes of fishes from European seas.

The 16S sequences showed the lowest mean genetic p-distances at all taxonomic levels, from species to orders, while the highest values were observed for cyt *b*, except at the species level (Table 2). The p-distance frequency distribution of the three markers did not showed any evidence for a barcoding gap (Fig. 2), which is an ideal case where the genetic divergence among nucleotide sequences at within- and between-species levels do not overlap [46]. However, in cyt *b*, the overlap of p-distance variation at within- and between-species levels was strongly reduced.

**Table 2:** Summary of genetic p-distances (%) within different taxonomic levels.

	16S		cyt *b*		COI	
comparison within	Mean p-distance	SE	Mean p-distance	SE	Mean p-distance	SE
species	0.23	0.11	0.57	0.22	0.59	0.17
genera	2.66	0.46	7.72	0.86	3.96	0.52
families	4.35	0.53	10.94	0.91	9.42	0.86
orders	10.78	0.90	16.38	1.04	13.52	1.02

Values are calculated from partial sequences of the mitochondrial 16S rRNA (16S; n = 369), cytochrome *b* (cyt *b*; n = 212), and cytochrome oxidase subunit I (COI; n = 447) genes of 50 fish species from European seas.

**Figure 3:**
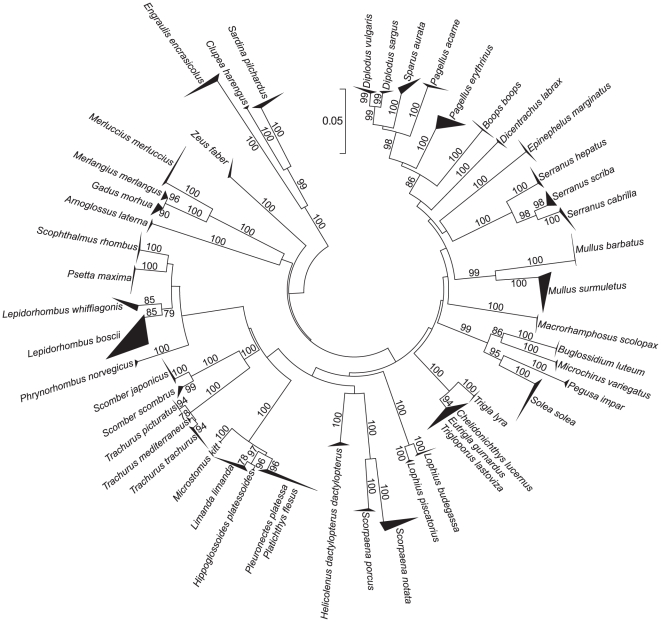
Phylogenetic analysis (16S). Neighbour Joining tree for partial sequences of the mitochondrial 16S rRNA gene of fishes from European seas. The number of sequences and their geographic origin for each species are given in Table 1 and Supporting Information Table S1. Bootstrap values based on 1000 replicates are indicated at branches.

**Figure 4:**
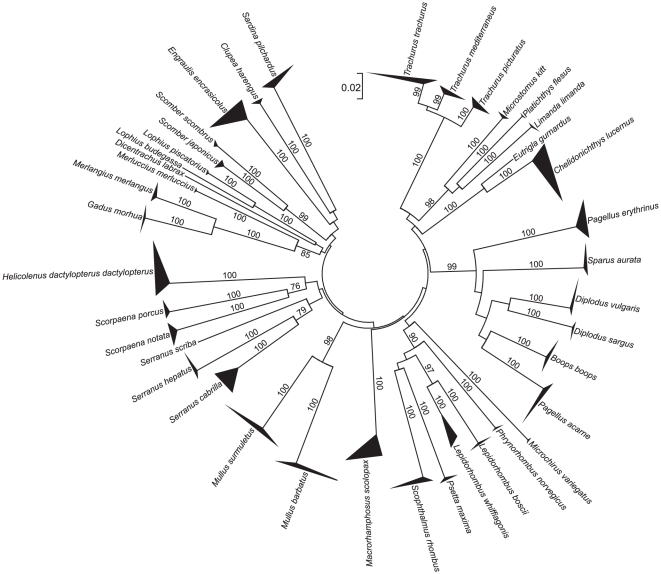
Phylogenetic analysis (cyt *b*). Neighbour Joining tree for partial sequences of the mitochondrial cytochrome *b* gene of fishes from European seas. The number of sequences and their geographic origin for each species are given in Table 1 and Supporting Information Table S1. Bootstrap values based on 1000 replicates are indicated at branches.

**Figure 5:**
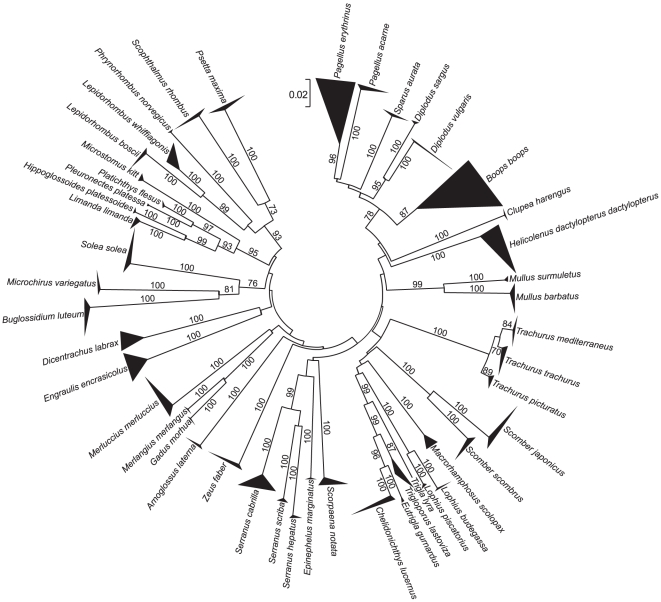
Phylogenetic analysis (COI). Neighbour Joining tree for partial sequences of the mitochondrial cytochrome oxidase subunit I gene of fishes from European seas. The number of sequences and their geographic origin for each species are given in Table 1 and Supporting Information Table S1. Bootstrap values based on 1000 replicates are indicated at branches.

All NJ trees resolved species-specific clades that were supported by high bootstrap values (Fig. 3, Fig. 4, and Fig. 5), except for the 16S tree that was unable to separate the nucleotide sequences of the closely related flatfish species *Pleuronectes platessa* and *Platichthys flesus* and of the gurnards *Chelidonichthys lucernus*, *Eutrigla gurnardus*, and *Trigloporus lastoviza* (Fig. 3).

### DNA microarray

**Figure 6:**
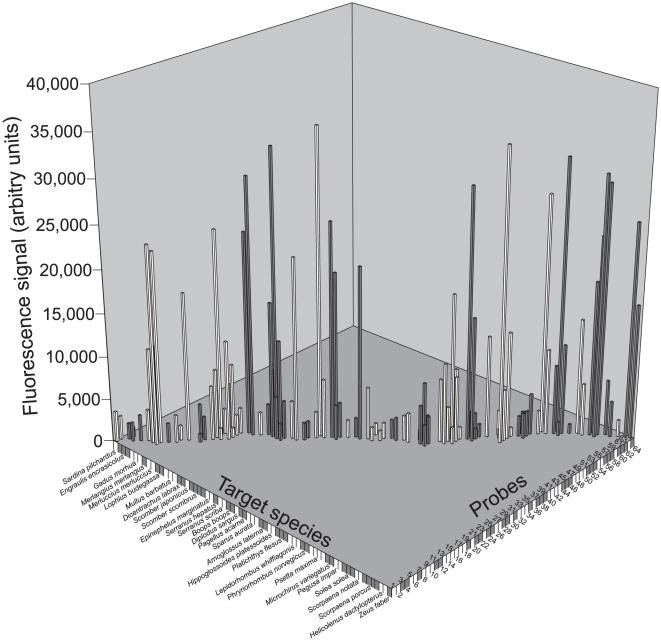
DNA microarray hybridisation experiments. Mean signal intensities of single target hybridisations to 64 oligonucleotide probes on a DNA microarray for the identification of 30 fish species from European seas. For numbers given to oligonucleotide probes refer to Table 3 and Supporting Information Table S2.

A total of 319 oligonucleotide probes (16S: 46; cyt *b*: 123; COI: 150) were designed for the 50 target species (Table 1, Supporting Information Table S1) and tested within 255 hybridisation experiments with 3 replicates each (data not shown). Several probes were not functional due to low signal intensities as well as false-positive or false-negative signals. A total of 64 probes unambiguously identified 30 target fish species (Table 3, Supporting Information Table S2, and Fig. 6). However, the portion of the *in silico* selected probes that gave successful hybridisation signals with target species was greatly variable among gene markers: 20 16S-probes for 15 species (43.5%), 31 cyt *b*-probes for 16 species (25.2%), and 13 COI-probes for 10 species (8.7%).

**Table 3:** Oligonucleotide probes for the identification of fish species from European seas.

No.	Species	Probe ID	Probe sequence (5′>3′)
1	*Sardina pilchardus*	Cytb_Sarpil_l25_p203	CACAGTTCTTCACCTGCTCTTCCTC
2	*Sardina pilchardus*	Cytb_Sarpil_l26_p170	CCACTTCTTGTTCCCATTCGTGATCG
3	*Engraulis encrasicolus*	COI_Engenc_l27_p182	CCTTCTCCTCTTAGCATCATCTGGTGT
4	*Engraulis encrasicolus*	Cytb_Engenc_l23_p194	TGCAGGTGTTACTATCCTTCACC
5	*Gadus morhua*	Cytb_Gadmor_l26_p362	CGCACCTAATTTACTCGGAGATCCAG
6	*Gadus morhua*	Cytb_Gadmor_l27_p351	CTCGCCCTCTTCGCACCTAATTTACTC
7	*Merlangius merlangus*	Cytb_Mermer_l23_p334	TTCTAGGCTTAACTGCTCTGGCC
8	*Merluccius merluccius*	Cytb_Mercmerc_l23_p325	CTCTGCTCCTTATCGCCCTAACA
9	*Merluccius merluccius*	Cytb_Mercmerc_l24_p252	GTAGGGCTCAACTCTGATGCAGAC
10	*Merluccius merluccius*	COI_Mercmerc_l23_p398	ACCCCTCTTTGTTTGATCCGTCC
11	*Lophius budegassa*	Cytb_Lopbud_l25_p194	CCTGGCAATAACCGTTATCCACCTC
12	*Lophius budegassa*	Cytb_Lopbud_l26_p325	CAGTCGTCTTAATTACGCTCACAGCC
13	*Dicentrarchus labrax*	16S_Diclab_l25_p202	GGGAGACTACCTTAATTACCCCTGG
14	*Dicentrarchus labrax*	16S_Diclab_l23_p236	AAAAGCTAAAGGTACCCCTCCCC
15	*Dicentrarchus labrax*	COI_Diclab_l25_p378	GCCATTTCCCAGTACCAAACTCCTT
16	*Dicentrarchus labrax*	Cytb_Diclab_l23_p199	GTGCCACAATACTACACCTCCTT
17	*Dicentrarchus labrax*	Cytb_Diclab_l27_p216	CTCCTTTTTCTTCATCAAACGGGCTCC
18	*Dicentrarchus labrax*	Cytb_Diclab_l27_p247	ACCCCTTAGGCCTTAACTCAGATGTAG
19	*Mullus barbatus*	16S_Mulbar_l25_p357	CTTCTGACCTACAAGATCCGGCCAA
20	*Scomber japonicus*	16S_Scojap_l23_p223	CCCCTAACAAGGGGCCAAACTTA
21	*Scomber scombrus*	Cytb_Scosco_l25_p324	GCCGTTCTCCTTATAGGCCTTACCT
22	*Scomber scombrus*	Cytb_Scosco_l25_p335	TATAGGCCTTACCTCCCTAGCACTC
23	*Epinephelus marginatus*	16S_Epimar_l24_p216	TAATACCCTCAACAACAGGACACG
24	*Serranus hepatus*	COI_Serhep_l26_p232	GAACTGTTTATCCGCCTTTAGCTGGT
25	*Serranus hepatus*	COI_Serhep_l27_p243	CCGCCTTTAGCTGGTAACTTAGCTCAC
26	*Serranus scriba*	COI_Serscr_l23_p233	AACGGTTTACCCACCACTTGCTG
27	*Serranus scriba*	COI_Serscr_l27_p428	TGCAGTTCTCCTACTTCTATCCCTTCC
28	*Boops boops*	16S_Booboo_l23_p314	AGCACCACACTCCTAAACCCAAG
29	*Boops boops*	16S_Booboo_l24_p241	CCTAGTGAATCCTGCTCTAATGTC
30	*Diplodus sargus*	Cytb_Dipsar_l23_p197	CGCCATAACCATGCTTCACCTCT
31	*Pagellus acarne*	16S_Pagaca_l23_p316	GTACTACACTCCCACATCCGAGA
32	*Sparus aurata*	16S_Spaaur_l23_p201	AGAACAGCTCACGTCAAACACCC
33	*Sparus aurata*	Cytb_Spaaur_l26_p187	TCGTCATTGCAGCCATAACCATACTG
34	*Sparus aurata*	Cytb_Spaaur_l27_p205	CCATACTGCATCTTCTGTTCCTCCATG
35	*Arnoglossus laterna*	COI_Arnlat_l17_p387	ATGTACCAAGCACCCCT
36	*Hippoglossoides platessoides*	COI_Hippla_l26_p236	CGTGTATCCTCCCCTTGCTGGAAATC
37	*Platichthys flesus*	Cytb_Plafle_l23_p250	CCACAGGGCTAAACTCAGACTCT
38	*Platichthys flesus*	Cytb_Plafle_l23_p328	TTCTCCTTACTGCACTGGCTTCG
39	*Platichthys flesus*	Cytb_Plafle_l25_p197	GGCCGCAACAGTAATTCACCTACTC
40	*Lepidorhombus whiffiagonis*	16S_Lepwhi_l24_p323	CCCCACCAACTCCTCCAAACTAGA
41	*Lepidorhombus whiffiagonis*	COI_Lepwhi_l19_p370	AACCCGCTACTGTCACCAT
42	*Lepidorhombus whiffiagonis*	COI_Lepwhi_l26_p362	CAACATAAAACCCGCTACTGTCACCA
43	*Lepidorhombus whiffiagonis*	Cytb_Lepwhi_l23_p312	CTCCTTGGCTTCGCAGTTCTCTT
44	*Phrynorhombus norvegicus*	16S_Phrnor_l23_p326	AGCACCCATCCCAATTACTCCTC
45	*Phrynorhombus norvegicus*	Cytb_Phrnor_l23_p328	TACTTCTGACGGCACTCACATCC
46	*Phrynorhombus norvegicus*	Cytb_Phrnor_l25_p311	CCTTCTTGGCTTCGCAGTACTTCTG
47	*Psetta maxima*	16S_Psemax_l25_p321	CCCCTTAACTCCTCCAAATGAGAGC
48	*Psetta maxima*	Cytb_Psemax_l23_p321	TTCGTCGTCCTCTTGACAGCACT
49	*Psetta maxima*	Cytb_Psemax_l23_p337	CAGCACTCGCAACCCTAGCTTTA
50	*Psetta maxima*	Cytb_Psemax_l25_p195	GCAGCAGTAACGGTTATTCACCTCC
51	*Microchirus variegatus*	Cytb_Micvar_l23_p343	TGGCAGCCCTAGCAATATTCTCC
52	*Microchirus variegatus*	Cytb_Micvar_l25_p312	CTCCTCGGATTCTCGATCCTACTCA
53	*Microchirus variegatus*	Cytb_Micvar_l27_p325	CGATCCTACTCATTTTATTGGCAGCCC
54	*Pegusa impar*	16S_Pegimp_l23_p206	GCCCGTCCCCAAACCTGAAATAA
55	*Pegusa impar*	16S_Pegimp_l26_p313	GCACTTTACCCCATTACTCTTTGCTC
56	*Solea solea*	16S_Solsol_l23_p202	TTCAGCCCGTCCCCAAATTCTAA
57	*Solea solea*	16S_Solsol_l25_p321	CCCTTCACTCCCTGCTCTTAGAAAC
58	*Solea solea*	COI_Solsol_l25_p191	TCTCACCTCATCCGTTGTTGAAGCC
59	*Scorpaena notata*	16S_Sconot_l25_p241	CTGGTGGACCTCTTCCCTAATGTCT
60	*Scorpaena porcus*	16S_Scopor_l26_p209	CCATGTCACTAACCCTTTGATACAGG
61	*Scorpaena porcus*	16S_Scopor_l24_p312	GGCACACCCGTTCCTTCAATTAAG
62	*Scorpaena porcus*	Cytb_Scopor_l25_p332	CCTTCTTGGCCTTACAATACTCGCG
63	*Helicolenus dactylopterus dactylopterus*	COI_Heldac_l19_p374	CCCAGCGATCTCTCAATAC
64	*Zeus faber*	16S_Zeufab_l26_p187	GAGCTTTAGACCTAATGCAGTCCACG

Probe ID: 16S, Cytb, and COI indicate the mitochondrial 16S rRNA, cytochrome *b*, and cytochrome oxidase subunit I marker genes, respectively; the number following “l” is the length of the oligonucleotide probe and the number after “p” the position in the target sequence alignment. For details see Supporting Information Table S2.

Overall, the signal intensity was highly variable among individuals used in the hybridisation experiments and among probes of the three gene markers, ranging from 1,004 to 35,273 a.u.. (1) Some probes showed a large variation in signal intensity when PCR products of different individuals of the target species were hybridised on the microarray. For instance, in cod (*Gadus morhua*) the values for different specimens showed a 5–6 fold difference. (2) Among gene markers, the median value of the hybridisation signals obtained with the 16S-probes was much higher (11,915 a.u.) than those of the COI (3,027 a.u.) and cyt *b* probes (3,014 a.u.). However, this general pattern was not observed in all species. For example, the COI-probes of the European seabass (*Dicentrarchus labrax*) showed higher values than the cyt *b* and 16S probes (Table 3, Supporting Information Table S2, and Fig. 6). (3) Finally, additional variation among probes also resulted from the lack of positive hybridisation signals of some probes in some specimens of ten target species (i.e. *Engraulis encrasicolus*, *Merluccius merluccius*, *Dicentrarchus labrax*, *Serranus scriba*, *Sparus aurata*, *Platichthys flesus*, *Lepidorhombus whiffiagonis*, *Psetta maxima*, *Pegusa impar*, and *Solea solea*). However, for these species, at least one designed probe showed a clear positive signal (Fig. 6).

**Figure 7:**
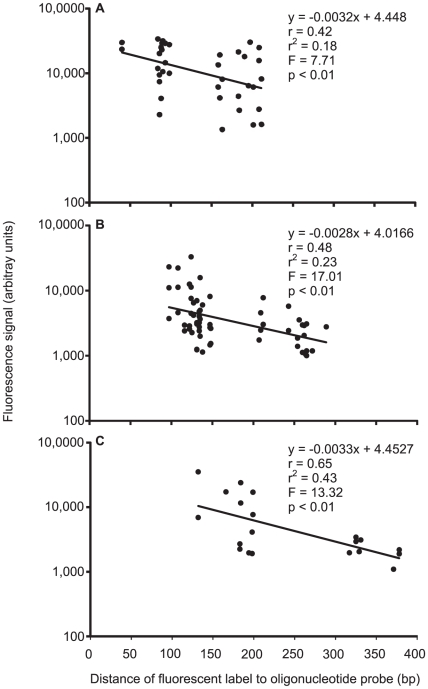
Position of label effect. Relationship of signal intensity and distance of fluorescent label to oligonucleotide probes based on partial sequences of mitochondrial (A) 16S rRNA, (B) cytochrome *b*, and (C) cytochrome oxidase subunit I genes of fishes from European seas.

The hybridisation signal intensity decreased as the distance between the binding site and the fluorescent label in the oligonucleotide probe increased (Fig. 7). This “position of label” (POL) effect [56], [57] was significant for all markers (p<0.01) and higher in the COI probes (r = 0.65) than in the cyt *b* (r = 0.48) and 16S probes (r = 0.42).

## Discussion

### DNA Barcoding

All three mitochondrial sequence markers were useful for the identification of the 50 target species (Fig. 3, Fig. 4, and Fig. 5). However, all of them had some limitations. Even though mean genetic p-distances were different at the multiple taxonomic levels suggesting the existence of a “barcoding gap” (Table 2), the frequency distribution of p-distances did not support the presence of such a gap (Fig. 2). This consistently supports the issue that mean values of genetic distances exaggerate the size of the “barcoding gap” [58]. In our data, the extent of overlap between genetic variation observed at within- and between-species levels was different among markers, with the largest overlap shown by 16S. The lack of a “barcoding gap” in COI was also observed in a comprehensive study on publicly available sequences of marine and freshwater fishes [59], available from the Barcoding of Life Database (BOLD) [60]. A limitation of the 16S marker was the lack of resolution in the species separation of related flatfish (*P. platessa* and *P. flesus*) and gurnard species (*C. lucernus*, *E. gurnardus*, and *T. lastoviza*). Even though the two flatfish species and other gurnards are known to hybridise [61], [62], the failing of 16S in discriminating them is not likely caused by introgression, because the same species and specimens were clearly separated by COI. Therefore, it is more reasonable to explain such lack of resolution with the rather low mutation rate in 16S. So far only six fish species were potentially affected by introgression in DNA barcoding studies [25], [63] and it is rather a minor problem in applying mtDNA in fish species identification [26].

The results clearly show that Numts, which may interfere in mtDNA-based species identification, are of no concern in this study. Numts are copies of mitochondrial genes or fragments of them that have been transferred to the nuclear genome. Since most Numts are smaller than 400 bp [64], it is very unlikely that they can amplify with the primer sets used in this study. Moreover, Numts are not expressed and consequently they can have a much higher mutation rate that is likely to lead to stop codons, gaps, or radical changes in the amino acid sequence in protein coding genes, which can be easily detected with bioinformatic analysis. Overall, Numts are also rather of little concern in applying mtDNA for species identification [26] and were not considered in this study as potential artefact.

### DNA Microarray

*In silico* probe design yielded a high number of potentially functional probes, but hybridisation experiments showed that most of them did not perform as expected from bioinformatic computations. Such a discrepancy between the performance exhibited by probes *in silico* and experimental hybridisations has already been reported by other studies for DNA [65] and RNA [66], suggesting that dynamics and processes of the hybridisation are still not understood. The unpredictable performance of probes in the microarray experiments lead to high variation of hybridisation signals. The median value of 16S hybridisation signals was four times higher than those obtained with cyt *b* and COI probes. However, most functional oligonucleotide probes were based on cyt *b* sequences and they also detected the highest number of target species (Table 3, Supporting Information Table S2). Even though most potentially functional probes could be designed based on COI, more than 90% had to be rejected due to cross-hybridisations and lack of signal in hybridisation experiments. In comparison, the rejection rates of 16S (56%) and cyt *b* (74%) probes were lower. Comparatively, in *Penicillium* approximately 60% of COI-based probes developed for species detection were rejected [65]. Overall, these results indicate that all oligonucleotide probes have to be tested intensively by hybridisation experiments to evaluate their functionality in species identification, preferably with several individuals of the target species together with a high number of non-target species. The *in silico* study on the effectiveness of mammalian COI and cyt *b* sequences for probe design suggested that both genes yield a high number of probes [67]. However, since the behaviour of oligonucleotide probes in hybridisation experiments cannot be predicted, *in silico* results should be handled with caution. The present study rather suggests that COI and cyt *b* are not well suited for probe design in fish species and similar findings were reported for the COI in fungi [65]. Sequences of ribosomal genes (e.g. 16S) seem to be more suitable for the design of functional probes in the studied fish species. This should be related to the secondary structure of the rDNA, showing single stranded linear DNA, single stranded loops, and double stranded stems. The loop region is characterised by a high insertion/deletion polymorphism (indel), which is a valuable feature making these sequences suitable for the design of highly specific oligonucleotide probes [42]. On the contrary, the disadvantage of 16S rDNA sequences is the lack of discrimination power among closely related species. However, this problem can be overcome by analysing in parallel other gene markers.

Absolute signal intensities were very heterogeneous in this study, the maximum value being 35-fold higher than the minimum value (Fig. 6). Great variation in signal intensities commonly affects DNA microarray hybridisation experiments (e.g., [68]–[72]). On the one hand, variation in signal intensity given by a certain oligonucleotide probe can occur among different experimental replicates and this might be related to differences in the quality of slides or solutions used for the hybridisation and washing steps. It is also reported that increased atmospheric ozone concentrations cause the oxidation of Cy5, hence decreasing fluorescence signal intensities [73], [74]. On the other hand, large differences in signal intensities among oligonucleotide probes might be related to the number and position of mismatches. Additionally, there are also sequence specific differences [75]. This study has also shown differences of the mean signal intensity among the three markers. While oligonucleotide probes based on cyt *b* and COI showed almost identical mean values of signal intensity, the mean value for 16S-probes was about four times higher. This might be explained by the secondary structure of the target DNA. In 16S, all oligonucleotide probes bind to the variable regions *j* and *l* [42], which represent large single-stranded loops. Therefore, the binding sites in the 16S target DNA are freely accessible for the oligonucleotide probes. In contrast, secondary structures of the protein coding cyt *b* and COI DNA fragments might hamper access of the probes to the binding sites in the target DNA.

The position of label relative to the target DNA-probe duplex might cause variation of the signal intensities among different oligonucleotide probes. Highest signal intensities are given by probes with a low distance between the fluorescent label and the binding site. Signal intensity decreases with increasing distance [56], [57]. The highest correlation was found in COI, followed by cyt *b* and 16S (Fig. 5). This was due to the fact that the maximum distance of the binding site to the fluorescence label is only about 200 bp in 16S, while it is almost 300 bp in cyt *b* and almost 400 bp in COI. Our results support that the 16S fragment is the most suited marker for microarray probe design, compared to cyt *b* and COI fragments.

### Conclusions

The present study showed that the investigated mitochondrial sequence markers perform differently in DNA barcoding and microarray analyses for the identification of fish species. While cyt *b* and COI are equally well suited for the sequence based species identification of fishes, 16S has drawbacks in discriminating closely related species. In contrast, 16S-probes performed appreciably better than probes based on cyt *b* and COI in DNA microarray hybridisation experiments. Oligonucleotide probes based on 16S showed a lower rejection rate after hybridisation experiments, higher mean signal intensity, and weaker position of lable (POL) effect. Therefore, 16S sequences can be recommended for designing oligonucleotide probes for fish species identification based on DNA microarrays. In order to allow the discrimination of closely related species, additional markers, such as cyt *b* or a nuclear gene would be helpful. Unfortunately, COI was not suitable for the design of oligonucleotide probes for the target species, discouraging the utilisation of the huge number of COI barcode sequences in the Barcoding of Life Database (BOLD) [60] as a data source for the development of DNA microarrays for the identification of fish species.

This study has shown that mitochondrial sequence markers can be useful tools for the identification of European marine fishes. Species assignment is very important in the context of fisheries research, fisheries control, and consumer protection. The development of the described DNA microarray for the identification of 30 fish species represents the next step towards an automated and easy-to-handle assay that can be applied in ichthyoplankton surveys, by companies involved in fish trade as well as authorities concerned with fisheries control and consumer protection.

## Supporting Information

Table S1 Sequences utilised for the DNA barcoding approach. Abbreviations: 16S: 16S rRNA gene, cyt *b*: cytochrome b gene, COI: cytochrome oxidase subunit I gene, O: order, C: Clupeiformes, G: Gadiformes, L: Lophiiformes, P: Perciformes, Pl: Pleuronectiformes, S: Scorpaeniformes, Sy: Syngnathiformes, Z: Zeiformes, NA: Northeastern Atlantic, NS: North Sea, B: Baltic, BB: Bay of Biscay, WM: Western Mediterranean, CM: Central Mediterranean, EM: Eastern Mediterranean, and BS: Black Sea. No number in cell = 0.
(0.22 MB DOC)

Table S2 Oligonucleotide probes for the identification of fish species from European seas. Probe ID: 16S, Cytb, and COI indicate the mitochondrial 16S rRNA, cytochrome *b*, and cytochrome oxidase subunit I marker genes, respectively; the number following “l” is the length of the oligonucleotide probe and the number after “p” the position in the target sequence alignment. Oligo mfe: minimal free energy of the secondary structure of the oligonucleotide; Dimer mfe: minimal free energy of the dimer of two identical oligonucleotide molecules. Values for mfe are given in kcal/mol. Mean fluorescence signal intensity as shown in Fig. 6 and its standard deviation (SD) is given in arbitrary units. Please note that some probes have been hybridised with several specimens of the target species.
(0.23 MB DOC)
