# Supplementary material for: Identifying Fishes through DNA Barcodes and Microarrays
Source: PLoS One. 2010 Sep 7;5(9):e12620. doi: 10.1371/journal.pone.0012620 (PMC2935389; doi:10.1371/journal.pone.0012620)
Supplement: Table S1 — Sequences utilised for the DNA barcoding approach. Abbreviations: 16S: 16S rRNA gene, cyt b: cytochrome b gene, COI: cytochrome oxidase subunit I gene, O: order, C: Clupeiformes, G: Gadiformes, L: Lophiiformes, P: Perciformes, Pl: Pleuronectiformes, S: Scorpaeniformes, Sy: Syngnathiformes, Z: Zeiformes, NA: Northeastern Atlantic, NS: North Sea, B: Baltic, BB: Bay of Biscay, WM: Western Mediterranean, CM: Central Mediterranean, EM: Eastern Mediterranean, and BS: Black Sea. No number in cell = 0. (0.22 MB DOC) [file pone.0012620.s001.doc]

**Supporting Information Table 1. Sequences utilised for the DNA barcoding approach.**

Abbreviations: 16S: 16S rRNA gene, cyt *b*: cytochrome *b* gene, COI: cytochrome oxidase subunit I gene, O: order, C: Clupeiformes, G: Gadiformes, L: Lophiiformes, P: Perciformes, Pl: Pleuronectiformes, S: Scorpaeniformes, Sy: Syngnathiformes, Z: Zeiformes, NA: Northeastern Atlantic, NS: North Sea, B: Baltic, BB: Bay of Biscay, WM: Western Mediterranean, CM: Central Mediterranean, EM: Eastern Mediterranean, and BS: Black Sea. No number in cell = 0.

|  |  |  | **16S** | | | | | | | | | **cyt *b*** | | | | | | | | | **COI** | | | | | | | | |  |
| --- | --- | --- | --- | --- | --- | --- | --- | --- | --- | --- | --- | --- | --- | --- | --- | --- | --- | --- | --- | --- | --- | --- | --- | --- | --- | --- | --- | --- | --- | --- |
| **Species** | **Family** | **O** | **NA** | **NS** | **B** | **BB** | **WM** | **CM** | **EM** | **BS** | **∑** | **NA** | **NS** | **B** | **BB** | **WM** | **CM** | **EM** | **BS** | **∑** | **NA** | **NS** | **B** | **BB** | **WM** | **CM** | **EM** | **BS** | **∑** | **Total** |
| *Clupea harengus* | Clupeidae | C |  |  | 2 |  |  |  |  |  | **2** |  | 3 |  |  |  |  |  |  | **3** |  | 2 | 3 |  |  |  |  |  | **5** | **10** |
| *Sardina pilchardus* | Clupeidae | C |  |  |  | 3 | 3 |  | 5 |  | **11** |  |  |  | 3 | 2 |  | 2 |  | **7** |  |  |  |  |  |  |  |  |  | **18** |
| *Engraulis encrasicolus* | Engraulidae | C |  |  |  | 3 | 2 |  | 6 |  | **11** |  |  |  | 3 | 2 |  | 3 |  | **8** |  |  | 2 | 3 | 5 |  | 2 |  | **12** | **31** |
| *Gadus morhua* | Gadidae | G | 1 | 1 |  | 3 |  |  |  |  | **5** | 1 | 1 |  | 3 |  |  |  |  | **5** | 1 | 2 |  | 2 |  |  |  |  | **5** | **15** |
| *Merlangius merlangus* | Gadidae | G |  |  |  | 4 |  |  |  |  | **4** |  | 3 |  | 1 |  |  |  |  | **4** | 2 | 1 |  |  |  |  |  | 1 | **4** | **12** |
| *Merluccius merluccius* | Merlucciidae | G |  |  |  | 2 | 3 | 3 | 6 |  | **14** |  |  |  |  |  | 2 |  |  | **2** |  | 1 |  | 3 | 5 | 5 | 3 | 2 | **19** | **35** |
| *Lophius budegassa* | Lophiidae | L |  |  |  | 3 |  |  | 5 |  | **8** |  |  |  | 1 |  |  |  |  | **1** |  |  |  | 3 |  | 2 | 1 |  | **6** | **15** |
| *Lophius piscatorius* | Lophiidae | L |  |  |  | 2 | 2 |  |  |  | **4** |  |  |  |  | 2 |  |  |  | **2** |  |  |  |  | 3 |  |  |  | **3** | **9** |
| *Trachurus mediterraneus* | Carangidae | P |  |  |  |  | 2 |  | 1 |  | **3** |  |  |  |  | 3 | 3 |  |  | **6** |  |  |  |  | 9 |  | 2 | 1 | **12** | **21** |
| *Trachurus picturatus* | Carangidae | P |  |  |  | 1 |  | 1 | 3 |  | **5** |  |  |  | 2 |  |  | 3 |  | **5** |  |  |  | 2 |  | 3 | 1 |  | **6** | **16** |
| *Trachurus trachurus* | Carangidae | P |  | 1 |  |  |  | 1 | 5 | 2 | **9** |  | 3 |  | 2 |  | 2 | 6 |  | **13** |  | 2 | 2 |  | 1 |  | 3 | 4 | **12** | **34** |
| *Dicentrarchus labrax* | Moronidae | P |  |  |  | 3 | 2 |  |  |  | **5** |  |  |  | 1 |  |  |  |  | **1** |  |  |  | 2 | 1 |  | 2 | 3 | **8** | **14** |
| *Mullus barbatus* | Mullidae | P |  |  |  |  | 3 | 2 | 5 |  | **10** |  |  |  |  |  | 6 | 6 |  | **12** |  |  |  |  |  | 5 | 5 | 2 | **12** | **34** |
| *Mullus surmuletus* | Mullidae | P |  | 2 |  | 5 | 3 | 3 | 1 |  | **14** |  | 3 |  | 5 |  |  | 5 |  | **13** |  | 1 |  |  | 1 |  | 1 |  | **3** | **30** |
| *Scomber japonicus* | Scombridae | P |  |  |  | 2 | 2 | 1 | 3 | 1 | **9** |  |  |  | 3 |  |  |  |  | **3** |  |  |  | 3 | 6 | 2 | 3 | 3 | **17** | **29** |
| *Scomber scombrus* | Scombridae | P |  |  |  | 3 |  |  | 1 |  | **4** |  | 1 |  |  |  | 1 |  |  | **2** |  | 3 |  | 2 | 2 |  | 2 | 2 | **11** | **17** |
| *Epinephelus marginatus* | Serranidae | P |  |  |  |  | 5 |  | 3 |  | **8** |  |  |  |  |  |  |  |  |  |  |  |  |  | 4 |  | 1 |  | **5** | **13** |
| *Serranus cabrilla* | Serranidae | P |  |  |  |  | 3 | 1 | 4 |  | **8** |  |  |  |  | 3 |  | 3 |  | **6** |  |  |  |  | 12 |  | 3 |  | **15** | **29** |
| *Serranus hepatus* | Serranidae | P |  |  |  |  | 3 | 2 | 3 |  | **8** |  |  |  |  | 2 |  | 4 |  | **6** |  |  |  |  | 5 |  |  |  | **5** | **19** |
| *Serranus scriba* | Serranidae | P |  |  |  |  | 4 | 1 | 1 |  | **6** |  |  |  |  |  |  | 1 |  | **1** |  |  |  |  | 4 |  | 1 |  | **5** | **12** |
| *Boops boops* | Sparidae | P |  |  |  |  | 3 |  | 6 |  | **9** |  |  |  |  | 3 | 2 | 2 |  | **7** |  |  |  | 3 | 10 | 2 | 5 | 3 | **23** | **39** |
| *Diplodus sargus* | Sparidae | P |  |  |  | 1 |  |  | 4 |  | **5** |  |  |  |  |  |  | 4 |  | **4** |  |  |  |  | 1 |  | 3 |  | **4** | **13** |
| *Diplodus vulgaris* | Sparidae | P |  |  |  | 2 | 4 |  | 2 |  | **8** |  |  |  |  | 6 |  | 1 |  | **7** |  |  |  | 6 | 9 |  | 7 |  | **22** | **37** |
| *Pagellus acarne* | Sparidae | P |  |  |  | 2 | 3 |  | 3 |  | **8** |  |  |  | 3 | 3 |  | 3 |  | **9** |  |  |  | 2 | 5 |  | 5 |  | **12** | **29** |
| *Pagellus erythrinus* | Sparidae | P |  |  |  | 1 | 2 |  | 7 |  | **10** |  |  |  | 2 | 3 | 1 | 1 |  | **7** |  |  |  | 3 | 6 |  | 6 |  | **15** | **32** |
| *Sparus aurata* | Sparidae | P |  |  |  | 1 | 3 |  | 3 |  | **7** |  |  |  |  | 3 |  | 3 |  | **6** |  |  |  | 3 | 5 |  | 3 |  | **11** | **24** |
| *Arnoglossus laterna* | Bothidae | Pl |  | 4 |  |  |  |  | 1 |  | **5** |  |  |  |  |  |  |  |  |  |  | 3 | 2 |  |  | 1 | 2 |  | **11** | **13** |
| *Hippoglossoides platessoides* | Pleuronectidae | Pl |  | 2 |  |  |  |  |  |  | **2** |  |  |  |  |  |  |  |  |  |  | 1 | 2 |  |  |  |  |  | **3** | **5** |
| *Limanda limanda* | Pleuronectidae | Pl |  | 9 | 2 |  |  |  |  |  | **11** | 3 |  |  |  |  |  |  |  | **3** | 3 | 1 | 2 |  |  |  |  |  | **6** | **20** |
| *Microstomus kitt* | Pleuronectidae | Pl |  | 2 |  |  |  |  |  |  | **2** | 2 | 1 |  |  |  |  |  |  | **3** | 2 | 2 |  |  |  |  |  |  | **4** | **9** |
| *Platichthys flesus* | Pleuronectidae | Pl |  | 7 | 1 | 2 |  |  |  | 1 | **11** |  |  |  | 2 |  |  |  |  | **2** |  | 2 | 2 |  |  |  |  |  | **4** | **17** |
| *Pleuronectes platessa* | Pleuronectidae | Pl |  | 7 | 2 |  |  |  |  |  | **9** |  |  |  |  |  |  |  |  |  |  | 1 | 1 |  |  |  |  |  | **2** | **11** |
| *Lepidorhombus boscii* | [Scophthalmidae](http://filaman.ifm-geomar.de/Summary/FamilySummary.cfm?ID=515) | Pl |  | 11 |  |  |  |  | 1 |  | **12** |  |  |  | 1 |  | 4 |  |  | **5** |  |  |  |  |  | 6 | 1 | 2 | **9** | **26** |
| *Lepidorhombus whiffiagonis* | [Scophthalmidae](http://filaman.ifm-geomar.de/Summary/FamilySummary.cfm?ID=515) | Pl |  | 5 |  |  |  |  |  |  | **5** |  |  |  | 2 |  |  | 1 |  | **3** |  |  |  |  | 1 | 1 | 5 | 1 | **8** | **16** |
| *Phrynorhombus norvegicus* | Scophthalmidae | Pl |  | 3 |  |  |  |  |  |  | **3** |  | 3 |  |  |  |  |  |  | **3** |  | 3 |  |  |  |  |  |  | **3** | **9** |
| *Psetta maxima* | Scophthalmidae | Pl |  | 4 |  | 3 | 2 |  |  |  | **9** |  | 1 |  | 3 |  |  |  |  | **4** |  | 3 | 3 | 3 | 3 |  |  | 3 | **15** | **28** |
| *Scophthalmus rhombus* | Scophthalmidae | Pl |  | 4 |  | 3 | 2 |  |  |  | **9** |  | 2 |  | 6 |  |  |  |  | **8** |  | 1 | 3 | 6 | 2 |  |  | 1 | **13** | **30** |
| *Buglossidium luteum* | Soleidae | Pl |  | 1 |  | 1 | 3 |  |  |  | **5** |  |  |  |  |  |  |  |  |  |  | 3 |  | 2 | 5 | 3 |  |  | **13** | **18** |
| *Microchirus variegatus* | Soleidae | Pl |  |  |  | 1 | 2 | 1 |  |  | **4** |  |  |  |  |  | 2 |  |  | **2** |  |  |  | 2 | 5 | 2 |  |  | **9** | **15** |
| *Pegusa impar* | Soleidae | Pl |  |  |  |  | 3 |  |  |  | **3** |  |  |  |  |  |  |  |  |  |  |  |  |  |  |  |  |  |  | **3** |
| *Solea solea* | Soleidae | Pl |  | 6 | 2 | 3 | 3 |  | 1 |  | **15** |  |  |  |  |  |  |  |  |  |  | 3 | 3 | 3 | 6 |  | 3 |  | **18** | **33** |
| *Scorpaena notata* | Scorpaenidae | S |  |  |  |  | 5 |  | 6 |  | **11** |  |  |  |  | 5 |  |  |  | **5** |  |  |  |  | 5 |  | 5 |  | **10** | **26** |
| *Scorpaena porcus* | Scorpaenidae | S |  |  |  | 3 |  |  | 3 | 2 | **8** |  |  |  | 2 |  |  | 2 |  | **4** |  |  |  |  |  |  |  |  |  | **12** |
| *Helicolenus dactylopterus dactylopterus* | Sebastidae | S |  |  |  | 2 | 1 |  | 6 |  | **9** |  |  |  | 3 | 2 | 4 | 1 |  | **10** |  |  |  | 3 | 5 | 6 | 6 |  | **20** | **39** |
| *Chelidonichthys lucernus* | Triglidae | S |  | 1 |  | 3 |  |  | 6 |  | **10** |  | 3 |  | 2 | 4 |  | 2 |  | **11** |  | 3 |  | 3 | 4 |  | 3 | 3 | **16** | **37** |
| *Eutrigla gurnardus* | Triglidae | S |  |  |  | 2 | 1 |  |  |  | **3** |  |  |  |  | 1 |  |  |  | **1** |  |  |  | 1 |  | 1 |  |  | **2** | **6** |
| *Trigla lyra* | Triglidae | S |  |  |  | 3 |  |  | 3 |  | **6** |  |  |  |  |  |  |  |  |  |  |  |  |  |  | 1 |  |  | **1** | **7** |
| *Trigloporus lastoviza* | Triglidae | S |  |  |  | 3 | 1 | 1 | 2 |  | **7** |  |  |  |  |  |  |  |  |  |  |  |  |  |  | 3 | 2 |  | **5** | **12** |
| *Macrorhamphosus scolopax* | Centriscidae | Sy |  |  |  |  | 2 |  | 5 |  | **7** |  |  |  | 1 | 3 |  | 4 |  | **8** |  |  |  |  | 5 |  | 1 |  | **6** | **21** |
| *Zeus faber* | Zeidae | Z |  |  |  | 3 | 3 |  | 2 |  | **8** |  |  |  |  |  |  |  |  |  |  | 3 |  | 3 | 4 | 2 | 3 |  | **15** | **23** |
|  |  |  | **1** | **70** | **9** | **73** | **80** | **17** | **113** | **6** | **369** | **6** | **24** |  | **51** | **47** | **27** | **57** |  | **212** | **8** | **41** | **25** | **63** | **143** | **45** | **91** | **31** | **447** | **1023** |
| Accession numbers: |  |  | FN687913-FN688280 | | | | | | | | | FN688281-FN688492 | | | | | | | | | FN688905-FN689348 | | | | | | | | |  |
