# Supplementary material for: Identifying Fishes through DNA Barcodes and Microarrays
Source: PLoS One. 2010 Sep 7;5(9):e12620. doi: 10.1371/journal.pone.0012620 (PMC2935389; doi:10.1371/journal.pone.0012620)
Supplement: Table S2 — Oligonucleotide probes for the identification of fish species from European seas. Probe ID: 16S, Cytb, and COI indicate the mitochondrial 16S rRNA, cytochrome b, and cytochrome oxidase subunit I marker genes, respectively; the number following “l” is the length of the oligonucleotide probe and the number after “p” the position in the target sequence alignment. Oligo mfe: minimal free energy of the secondary structure of the oligonucleotide; Dimer mfe: minimal free energy of the dimer of two identical oligonucleotide molecules. Values for mfe are given in kcal/mol. Mean fluorescence signal intensity as shown in Fig. 6 and its standard deviation (SD) is given in arbitrary units. Please note that some probes have been hybridised with several specimens of the target species. (0.23 MB DOC) [file pone.0012620.s002.doc]

**Supporting Information Table 2. Oligonucleotide probes for the identification of fish species from European seas.**

Probe ID: 16S, Cytb, and COI indicate the mitochondrial 16S rRNA, cytochrome *b*, and cytochrome oxidase subunit I marker genes, respectively; the number following “l” is the length of the oligonucleotide probe and the number after “p” the position in the target sequence alignment. Oligo mfe: minimal free energy of the secondary structure of the oligonucleotide; Dimer mfe: minimal free energy of the dimer of two identical oligonucleotide molecules. Values for mfe are given in kcal/mol. Mean fluorescence signal intensity as shown in Fig. 6 and its standard deviation (SD) is given in arbitrary units. Please note that some probes have been hybridised with several specimens of the target species.

| **No.** | **Species** | **Probe ID** | **Probe sequence (5'>3')** | **Length (bp)** | **Tm (°C)** | **GC (%)** | **Oligo mfe** | **Dimer mfe** | **Mean fluorescence signal in arbitrary units** | **SD** |
| --- | --- | --- | --- | --- | --- | --- | --- | --- | --- | --- |
| 1 | *Sardina pilchardus* | Cytb_Sarpil_l25_p203 | CACAGTTCTTCACCTGCTCTTCCTC | 25 | 82.65 | 52 | ≤ 0 | -3.16 | 3553 | 706 |
| 2 | *Sardina pilchardus* | Cytb_Sarpil_l26_p170 | CCACTTCTTGTTCCCATTCGTGATCG | 26 | 82.57 | 50 | ≤ 0 | 0.97 | 2778 | 587 |
| 3 | *Engraulis encrasicolus* | COI_Engenc_l27_p182 | CCTTCTCCTCTTAGCATCATCTGGTGT | 27 | 82.62 | 48 | ≤ 0 | -0.92 | 1895 | 405 |
|  |  |  |  |  |  |  |  |  | 2188 | 765 |
| 4 | *Engraulis encrasicolus* | Cytb_Engenc_l23_p194 | TGCAGGTGTTACTATCCTTCACC | 23 | 79.92 | 48 | ≤ 0 | -3.46 | 3079 | 526 |
|  |  |  |  |  |  |  |  |  | 1186 | 230 |
| 5 | *Gadus morhua* | Cytb_Gadmor_l26_p362 | CGCACCTAATTTACTCGGAGATCCAG | 26 | 81.60 | 50 | ≤ 0 | -2.69 | 3719 | 1324 |
|  |  |  |  |  |  |  |  |  | 11067 | 2482 |
|  |  |  |  |  |  |  |  |  | 23161 | 4644 |
| 6 | *Gadus morhua* | Cytb_Gadmor_l27_p351 | CTCGCCCTCTTCGCACCTAATTTACTC | 27 | 83.44 | 52 | ≤ 0 | 0.26 | 4595 | 1642 |
|  |  |  |  |  |  |  |  |  | 11249 | 3420 |
|  |  |  |  |  |  |  |  |  | 22246 | 4753 |
| 7 | *Merlangius merlangus* | Cytb_Mermer_l23_p334 | TTCTAGGCTTAACTGCTCTGGCC | 23 | 82.21 | 52 | ≤ 0 | -3.38 | 2267 | 740 |
| 8 | *Merluccius merluccius* | Cytb_Mercmerc_l23_p325 | CTCTGCTCCTTATCGCCCTAACA | 23 | 81.65 | 52 | ≤ 0 | 0.34 | 3227 | 787 |
| 9 | *Merluccius merluccius* | Cytb_Mercmerc_l24_p252 | GTAGGGCTCAACTCTGATGCAGAC | 24 | 82.25 | 54 | ≤ 0 | -2.81 | 1744 | 403 |
| 10 | *Merluccius merluccius* | COI_Mercmerc_l23_p398 | ACCCCTCTTTGTTTGATCCGTCC | 23 | 82.72 | 52 | ≤ 0 | -0.24 | 17284 | 9782 |
| 11 | *Lophius budegassa* | Cytb_Lopbud_l25_p194 | CCTGGCAATAACCGTTATCCACCTC | 25 | 82.47 | 52 | ≤ 0 | -2.50 | 1004 | 123 |
| 12 | *Lophius budegassa* | Cytb_Lopbud_l26_p325 | CAGTCGTCTTAATTACGCTCACAGCC | 26 | 82.16 | 50 | ≤ 0 | -1.14 | 4019 | 957 |
|  |  |  |  |  |  |  |  |  | 2759 | 409 |
| 13 | *Dicentrarchus labrax* | 16S_Diclab_l25_p202 | GGGAGACTACCTTAATTACCCCTGG | 25 | 81.14 | 52 | ≤ 0 | -3.71 | 6382 | 1941 |
| 14 | *Dicentrarchus labrax* | 16S_Diclab_l23_p236 | AAAAGCTAAAGGTACCCCTCCCC | 23 | 82.34 | 52 | ≤ 0 | -2.35 | 8097 | 2747 |
|  |  |  |  |  |  |  |  |  | 1351 | 327 |
| 15 | *Dicentrarchus labrax* | COI_Diclab_l25_p378 | GCCATTTCCCAGTACCAAACTCCTT | 25 | 82.66 | 48 | ≤ 0 | -0.18 | 11614 | 2572 |
|  |  |  |  |  |  |  |  |  | 23975 | 7062 |
| 16 | *Dicentrarchus labrax* | Cytb_Diclab_l23_p199 | GTGCCACAATACTACACCTCCTT | 23 | 80.18 | 48 | ≤ 0 | 0.49 | 2966 | 914 |
|  |  |  |  |  |  |  |  |  | 1124 | 223 |
| 17 | *Dicentrarchus labrax* | Cytb_Diclab_l27_p216 | CTCCTTTTTCTTCATCAAACGGGCTCC | 27 | 82.79 | 48 | ≤ 0 | 0.78 | 5753 | 866 |
|  |  |  |  |  |  |  |  |  | 2433 | 417 |
| 18 | *Dicentrarchus labrax* | Cytb_Diclab_l27_p247 | ACCCCTTAGGCCTTAACTCAGATGTAG | 27 | 82.31 | 48 | ≤ 0 | -2.79 | 7775 | 1589 |
|  |  |  |  |  |  |  |  |  | 3024 | 662 |
| 19 | *Mullus barbatus* | 16S_Mulbar_l25_p357 | CTTCTGACCTACAAGATCCGGCCAA | 25 | 83.58 | 52 | ≤ 0 | -1.86 | 23478 | 3135 |
|  |  |  |  |  |  |  |  |  | 29788 | 4964 |
| 20 | *Scomber japonicus* | 16S_Scojap_l23_p223 | CCCCTAACAAGGGGCCAAACTTA | 23 | 82.79 | 52 | ≤ 0 | -5.96 | 2679 | 415 |
| 21 | *Scomber scombrus* | Cytb_Scosco_l25_p324 | GCCGTTCTCCTTATAGGCCTTACCT | 25 | 83.10 | 52 | ≤ 0 | -2.81 | 3652 | 616 |
|  |  |  |  |  |  |  |  |  | 15798 | 4202 |
|  |  |  |  |  |  |  |  |  | 4984 | 1729 |
|  |  |  |  |  |  |  |  |  | 1995 | 645 |
| 22 | *Scomber scombrus* | Cytb_Scosco_l25_p335 | TATAGGCCTTACCTCCCTAGCACTC | 25 | 82.7 | 52 | ≤ 0 | -2.60 | 7584 | 1289 |
|  |  |  |  |  |  |  |  |  | 32986 | 9403 |
|  |  |  |  |  |  |  |  |  | 11329 | 3280 |
|  |  |  |  |  |  |  |  |  | 4464 | 472 |
| 23 | *Epinephelus marginatus* | 16S_Epimar_l24_p216 | TAATACCCTCAACAACAGGACACG | 24 | 79.88 | 46 | ≤ 0 | -0.44 | 4426 | 2789 |
|  |  |  |  |  |  |  |  |  | 21338 | 3811 |
| 24 | *Serranus hepatus* | COI_Serhep_l26_p232 | GAACTGTTTATCCGCCTTTAGCTGGT | 26 | 82.18 | 46 | ≤ 0 | -1.23 | 2052 | 519 |
| 25 | *Serranus hepatus* | COI_Serhep_l27_p243 | CCGCCTTTAGCTGGTAACTTAGCTCAC | 27 | 83.7 | 52 | ≤ 0 | -2.26 | 1971 | 401 |
| 26 | *Serranus scriba* | COI_Serscr_l23_p233 | AACGGTTTACCCACCACTTGCTG | 23 | 83.47 | 52 | ≤ 0 | -1.59 | 3111 | 662 |
| 27 | *Serranus scriba* | COI_Serscr_l27_p428 | TGCAGTTCTCCTACTTCTATCCCTTCC | 27 | 82.2 | 48 | ≤ 0 | -0.47 | 35273 | 5194 |
|  |  |  |  |  |  |  |  |  | 6931 | 1320 |
| 28 | *Boops boops* | 16S_Booboo_l23_p314 | AGCACCACACTCCTAAACCCAAG | 23 | 82.69 | 52 | ≤ 0 | -0.09 | 25067 | 8282 |
|  |  |  |  |  |  |  |  |  | 4082 | 799 |
| 29 | *Boops boops* | 16S_Booboo_l24_p241 | CCTAGTGAATCCTGCTCTAATGTC | 24 | 77.66 | 46 | ≤ 0 | -0.38 | 19134 | 6556 |
|  |  |  |  |  |  |  |  |  | 4154 | 640 |
| 30 | *Diplodus sargus* | Cytb_Dipsar_l23_p197 | CGCCATAACCATGCTTCACCTCT | 23 | 82.92 | 52 | ≤ 0 | 0.23 | 2049 | 504 |
|  |  |  |  |  |  |  |  |  |  |  |
| 31 | *Pagellus acarne* | 16S_Pagaca_l23_p316 | GTACTACACTCCCACATCCGAGA | 23 | 80.75 | 52 | ≤ 0 | -0.54 | 2298 | 771 |
|  |  |  |  |  |  |  |  |  | 20184 | 3757 |
| 32 | *Sparus aurata* | 16S_Spaaur_l23_p201 | AGAACAGCTCACGTCAAACACCC | 23 | 83.02 | 52 | ≤ 0 | -0.5 | 6130 | 1149 |
|  |  |  |  |  |  |  |  |  | 1590 | 273 |
| 33 | *Sparus aurata* | Cytb_Spaaur_l26_p187 | TCGTCATTGCAGCCATAACCATACTG | 26 | 82.7 | 52 | ≤ 0 | -2.60 | 1186 | 287 |
| 34 | *Sparus aurata* | Cytb_Spaaur_l27_p205 | CCATACTGCATCTTCTGTTCCTCCATG | 27 | 81.91 | 48 | ≤ 0 | 0.34 | 1393 | 286 |
|  |  |  |  |  |  |  |  |  | 1865 | 574 |
| 35 | *Arnoglossus laterna* | COI_Arnlat_l17_p387 | ATGTACCAAGCACCCCT | 17 | 78.19 | 53 | ≤ 0 | -0.23 | 2243 | 557 |
|  |  |  |  |  |  |  |  |  | 2705 | 403 |
| 36 | *Hippoglossoides platessoides* | COI_Hippla_l26_p236 | CGTGTATCCTCCCCTTGCTGGAAATC | 26 | 84.02 | 54 | ≤ 0 | -1.38 | 2943 | 1507 |
|  |  |  |  |  |  |  |  |  | 3433 | 2479 |
| 37 | *Platichthys flesus* | Cytb_Plafle_l23_p250 | CCACAGGGCTAAACTCAGACTCT | 23 | 81.66 | 52 | ≤ 0 | 0.82 | 4566 | 994 |
|  |  |  |  |  |  |  |  |  | 2464 | 433 |
| 38 | *Platichthys flesus* | Cytb_Plafle_l23_p328 | TTCTCCTTACTGCACTGGCTTCG | 23 | 81.66 | 52 | ≤ 0 | 0.82 | 1254 | 250 |
|  |  |  |  |  |  |  |  |  | 7004 | 929 |
|  |  |  |  |  |  |  |  |  | 3219 | 439 |
| 39 | *Platichthys flesus* | Cytb_Plafle_l25_p197 | GGCCGCAACAGTAATTCACCTACTC | 25 | 82.65 | 52 | ≤ 0 | -1.88 | 2914 | 863 |
| 40 | *Lepidorhombus whiffiagonis* | 16S_Lepwhi_l24_p323 | CCCCACCAACTCCTCCAAACTAGA | 24 | 83.61 | 54 | ≤ 0 | -0.07 | 7422 | 3384 |
|  |  |  |  |  |  |  |  |  | 9448 | 3492 |
| 41 | *Lepidorhombus whiffiagonis* | COI_Lepwhi_l19_p370 | AACCCGCTACTGTCACCAT | 19 | 80.55 | 53 | ≤ 0 | -0.11 | 4118 | 2310 |
|  |  |  |  |  |  |  |  |  | 1914 | 517 |
| 42 | *Lepidorhombus whiffiagonis* | COI_Lepwhi_l26_p362 | CAACATAAAACCCGCTACTGTCACCA | 26 | 82.27 | 46 | ≤ 0 | -0.14 | 17085 | 5747 |
|  |  |  |  |  |  |  |  |  | 7686 | 1738 |
| 43 | *Lepidorhombus whiffiagonis* | Cytb_Lepwhi_l23_p312 | CTCCTTGGCTTCGCAGTTCTCTT | 23 | 82.70 | 52 | ≤ 0 | 0.65 | 8131 | 1775 |
|  |  |  |  |  |  |  |  |  | 1465 | 310 |
| 44 | *Phrynorhombus norvegicus* | 16S_Phrnor_l23_p326 | AGCACCCATCCCAATTACTCCTC | 23 | 82.27 | 52 | ≤ 0 | -0.05 | 29119 | 7559 |
|  |  |  |  |  |  |  |  |  | 14528 | 2431 |
| 45 | *Phrynorhombus norvegicus* | Cytb_Phrnor_l23_p328 | TACTTCTGACGGCACTCACATCC | 23 | 81.90 | 52 | ≤ 0 | 0.70 | 3074 | 943 |
|  |  |  |  |  |  |  |  |  | 1235 | 291 |
| 46 | *Phrynorhombus norvegicus* | Cytb_Phrnor_l25_p311 | CCTTCTTGGCTTCGCAGTACTTCTG | 25 | 82.55 | 52 | ≤ 0 | -1.62 | 2626 | 897 |
|  |  |  |  |  |  |  |  |  | 1537 | 464 |
| 47 | *Psetta maxima* | 16S_Psemax_l25_p321 | CCCCTTAACTCCTCCAAATGAGAGC | 25 | 82.14 | 52 | ≤ 0 | -0.8 | 11915 | 2691 |
|  |  |  |  |  |  |  |  |  | 33823 | 5160 |
| 48 | *Psetta maxima* | Cytb_Psemax_l23_p321 | TTCGTCGTCCTCTTGACAGCACT | 23 | 83.67 | 52 | ≤ 0 | -2.44 | 1138 | 424 |
|  |  |  |  |  |  |  |  |  | 6017 | 1924 |
| 49 | *Psetta maxima* | Cytb_Psemax_l23_p337 | CAGCACTCGCAACCCTAGCTTTA | 23 | 82.66 | 52 | ≤ 0 | -1.33 | 2873 | 634 |
|  |  |  |  |  |  |  |  |  | 2571 | 783 |
|  |  |  |  |  |  |  |  |  | 12558 | 4277 |
| 50 | *Psetta maxima* | Cytb_Psemax_l25_p195 | GCAGCAGTAACGGTTATTCACCTCC | 25 | 82.65 | 52 | ≤ 0 | -3.16 | 1044 | 352 |
| 51 | *Microchirus variegatus* | Cytb_Micvar_l23_p343 | TGGCAGCCCTAGCAATATTCTCC | 23 | 82.49 | 52 | ≤ 0 | -2.79 | 2407 | 374 |
|  |  |  |  |  |  |  |  |  | 2947 | 718 |
| 52 | *Microchirus variegatus* | Cytb_Micvar_l25_p312 | CTCCTCGGATTCTCGATCCTACTCA | 25 | 82.49 | 52 | ≤ 0 | -2.79 | 2705 | 497 |
|  |  |  |  |  |  |  |  |  | 3003 | 964 |
| 53 | *Microchirus variegatus* | Cytb_Micvar_l27_p325 | CGATCCTACTCATTTTATTGGCAGCCC | 27 | 82.34 | 48 | ≤ 0 | -2.72 | 2409 | 258 |
|  |  |  |  |  |  |  |  |  | 4784 | 1026 |
| 54 | *Pegusa impar* | 16S_Pegimp_l23_p206 | GCCCGTCCCCAAACCTGAAATAA | 23 | 83.32 | 52 | ≤ 0 | -0.11 | 2780 | 791 |
| 55 | *Pegusa impar* | 16S_Pegimp_l26_p313 | GCACTTTACCCCATTACTCTTTGCTC | 26 | 80.87 | 46 | ≤ 0 | -0.36 | 27557 | 6294 |
|  |  |  |  |  |  |  |  |  | 10013 | 2515 |
| 56 | *Solea solea* | 16S_Solsol_l23_p202 | TTCAGCCCGTCCCCAAATTCTAA | 23 | 82.39 | 48 | ≤ 0 | -0.07 | 8130 | 1601 |
|  |  |  |  |  |  |  |  |  | 1623 | 487 |
| 57 | *Solea solea* | 16S_Solsol_l25_p321 | CCCTTCACTCCCTGCTCTTAGAAAC | 25 | 81.95 | 52 | ≤ 0 | -0.2 | 31591 | 6227 |
|  |  |  |  |  |  |  |  |  | 10559 | 2133 |
| 58 | *Solea solea* | COI_Solsol_l25_p191 | TCTCACCTCATCCGTTGTTGAAGCC | 25 | 84.09 | 52 | ≤ 0 | -0.71 | 1096 | 427 |
| 59 | *Scorpaena notata* | 16S_Sconot_l25_p241 | CTGGTGGACCTCTTCCCTAATGTCT | 25 | 82.91 | 52 | ≤ 0 | -2.51 | 13493 | 1460 |
|  |  |  |  |  |  |  |  |  | 6132 | 1327 |
| 60 | *Scorpaena porcus* | 16S_Scopor_l26_p209 | CCATGTCACTAACCCTTTGATACAGG | 26 | 80.09 | 46 | ≤ 0 | -0.64 | 18026 | 3250 |
|  |  |  |  |  |  |  |  |  | 30201 | 5097 |
| 61 | *Scorpaena porcus* | 16S_Scopor_l24_p312 | GGCACACCCGTTCCTTCAATTAAG | 24 | 81.57 | 50 | ≤ 0 | -0.81 | 22991 | 3855 |
|  |  |  |  |  |  |  |  |  | 29045 | 6178 |
| 62 | *Scorpaena porcus* | Cytb_Scopor_l25_p332 | CCTTCTTGGCCTTACAATACTCGCG | 25 | 82.59 | 52 | ≤ 0 | -2.74 | 6483 | 1669 |
|  |  |  |  |  |  |  |  |  | 4183 | 1393 |
| 63 | *Helicolenus dactylopterus dactylopterus* | COI_Heldac_l19_p374 | CCCAGCGATCTCTCAATAC | 19 | 75.94 | 53 | ≤ 0 | -0.35 | 1965 | 604 |
| 64 | *Zeus faber* | 16S_Zeufab_l26_p187 | GAGCTTTAGACCTAATGCAGTCCACG | 26 | 81.97 | 50 | ≤ 0 | -3.78 | 24847 | 3835 |
|  |  |  |  |  |  |  |  |  | 15691 | 2966 |
